# Identifying incident oral and pharyngeal cancer cases using Medicare claims

**DOI:** 10.1186/1472-6831-13-1

**Published:** 2013-01-01

**Authors:** Jonathan D Mahnken, John D Keighley, Douglas A Girod, Xueyi Chen, Matthew S Mayo

**Affiliations:** 1 Department of Biostatistics, The University of Kansas Medical Center, The University of Kansas Cancer Center, MSN 1026, 3901 Rainbow Blvd, Kansas City, KS, 66160, USA; 2 Department of Otolaryngology, The University of Kansas Medical Center, Kansas City, KS, USA; 3 Department of Biostatistics, The University of Kansas Medical Center, Kansas City, KS, USA

**Keywords:** Medicare, Oral cancer, Secondary data analysis, SEER

## Abstract

**Background:**

Baseline and trend data for oral and pharyngeal cancer incidence is limited. A new algorithm was derived using the Surveillance, Epidemiology, and End Results (SEER)-Medicare linked database to create an algorithm to identify incident cases of oral and pharyngeal cancer using Medicare claims.

**Methods:**

Using a split-sample approach, Medicare claims’ procedure and diagnosis codes were used to generate a new algorithm to identify oral and pharyngeal cancer cases and validate its operating characteristics.

**Results:**

The algorithm had high sensitivity (95%) and specificity (97%), which varied little by age group, sex, and race and ethnicity.

**Conclusion:**

Examples of the utility of this algorithm and its operating characteristics include using it to derive baseline and trend estimates of oral and pharyngeal cancer incidence. Such measures could be used to provide incidence estimates where they are lacking or to serve as comparator estimates for tumor registries.

## Background

The Surgeon General’s report on oral health in America stated that oral and craniofacial surveillance databases for diseases, health services, and health care utilization are limited or are lacking [1]. This report called for further development and validation of outcome measures [1]. The National Institute of Dental and Craniofacial Research (NIDCR) noted similar concerns [2], and described the need for baseline data in order to recognize trends over time, particularly for underserved populations and less common conditions among the general population [2].

Oral and pharyngeal (OP) cancers are diagnosed in approximately 30,000 individuals in the United States each year, and the annual mortality for OP cancer is approximately 7,500 [1-3]. A large portion of OP cases diagnosed are among individuals ages 65 years and older [4]. Despite the large number of new cases each year, OP cancer—relatively speaking—is a rare disease; so large population-based settings are often required to answer important research questions. Thus, we generated and validated an algorithm to identify incident OP cases based on Medicare claims.

Investigators have utilized Medicare claims for cancer and other health outcomes studies. Hospital, outpatient, and physician claims can be reconfigured into longitudinal databases. These can include diagnosis and procedure codes, cost of services provided, as well as the corresponding dates of these diagnoses and procedures.

Cooper et al. [5] studied the sensitivity of Medicare data to identify incident prostate, lung, colorectal, breast, pancreatic, and endometrial cancer cases. An algorithm by Freeman et al. [6] to identify incident breast cancer cases based on Medicare claims had high sensitivity (90%), and also measured the specificity and positive predictive value of their algorithm. McClish and Penberthy [7] used Medicare claims to quantify the number of missed cases in the Virginia cancer registry. Medicare claims created a unique opportunity for their work as it required data from three separate sources—the Virginia cancer registry, the Medicare Part A claims, and the Medicare Part B claims. Mandelblatt et al. [8] and Mandelblatt et al. [9] used the algorithm of Freeman et al. [6] to identify breast cancer cases to investigate treatments and perceptions of cancer treatment. Of particular interest to Mandelblatt et al. [8] were racial health disparities. In addition to the algorithm developed by Freeman et al. [6], Nattinger et al. [10] created a four-step algorithm to identify breast cancer cases among the Medicare population and improved the positive predictive value (≥89%) for identifying cases, while still retaining high levels of sensitivity and specificity.

In this study the SEER-Medicare linked database was used to derive an algorithm that identified incident OP cancer cases among the elderly using only Medicare claims. This algorithm may enable future studies to address research questions about OP cancer through secondary data analyses on Medicare claims. Individuals identified by the algorithm can simultaneously be linked to their medical records (Medicare claims) to assess health trajectories. Estimates of OP cancer incidence rates can also be derived using this algorithm for Medicare beneficiaries, a large population-base inclusive of many rural and other hard-to-reach populations in the United States.

## Methods

### Data sources

The SEER-Medicare linked database was used for this study. The Center for Medicare and Medicaid Services linked Surveillance, Epidemiology, and End Results (SEER) tumor registry data with Medicare claims and census information to create the SEER-Medicare linked database. These data contained information on individuals with cancer, identified as being cases in the SEER tumor registry, who also had Medicare insurance as indicated by Medicare enrollment records. Nearly all (97%) of the population ages 65 and older in the United States have Medicare health insurance coverage [11], which provides inpatient hospitalization, skilled nursing facility, home health, and hospice care (Part A) coverage. Most beneficiaries also have coverage for physician and outpatient care services (Part B) [11]. Demographic information was also available from Medicare enrollment files, including membership in Health Maintenance Organizations, or HMOs [11]. For this study, only inpatient (Part A) and physician and outpatient (Part B) Medicare claims were used. Previous investigations of the SEER-Medicare database indicated that approximately 93.6% of the cases in SEER tumor registry were also included in the SEER-Medicare database for subjects ages 65 years old and older [12].

The SEER-Medicare linked database consisted of two types of denominator files, the Patient Entitlement and Diagnosis Summary File (PEDSF) and Summarized Denominator File (SumDenom). The PEDSF file contained patient demographics collected by both SEER and the Social Security Administration. These included measures such as date of birth, race, ethnicity, county of residence, Medicare eligibility, HMO membership and date of death. Only individuals diagnosed in a SEER registry with cancer were included in the PEDSF file; thus tumor measures (e.g., cancer site, date of diagnosis, stage of tumor, etc.) from SEER were also included in these files. Our PEDSF file included patients in the SEER-Medicare linked database whose cancer diagnosis took place from 1973 to 2002. We obtained 100% files for subjects with OP cancers diagnosed during these years. Subjects with cancers other than OP cancer were not included in these analyses. The SumDenom file contained similar demographic information to the PEDSF, with its information gathered solely from the Social Security Administration. The information in the SumDenom file was a 5% sample of individuals living in SEER areas that had not linked to a SEER tumor registry. Information in our SumDenom file covered the years 1986 through 2004.

The Medicare claims portions of the SEER-Medicare linked database (inpatient, physician and outpatient files) were related to one another through a common, subject-specific identifier variable. The Medicare Provider Analysis Review (MedPAR) files contained hospital inpatient claims. These included ICD-9-CM diagnosis codes, ICD-9-CM procedure codes, as well as the corresponding dates of the diagnoses and procedures. The 100% Physician/Supplier (physician) files were a subset of the National Claims History files, and were referred to as the NCH files. These data included ICD-9-CM diagnosis codes and a CPT procedure code, along with the corresponding dates of these diagnoses and procedures. The Outpatient Standard Analytic (outpatient) files were also a subset of the National Claims History files, and were referred to as the OutSAF files. These data included ICD-9-CM diagnosis codes, ICD-9-CM procedure codes, and a CPT procedure code with accompanying dates.

### Study cohort

A total of 3,050 incident OP cancer cases with a date of diagnosis in the 2002 calendar year were identified. The lower age limit for inclusion was 66 years to allow for subjects aging into Medicare at age 65 to meet our prior coverage criteria, leaving 2,751 cases. To ensure complete information for each subject’s medical history, only subjects with coverage by Medicare (Parts A and B) but not by an HMO during the year before diagnosis through the year after diagnosis (or through death for subjects that died within a year of their diagnosis) were included. Following this exclusion, 1,807 OP cancer cases remained. For non-cancer controls, 472,293 subjects were identified from the SumDenom file as alive in 2002. A “pseudo-diagnosis date” was randomly assigned as a date in the 2002 calendar year. (Alternative random assignment strategies would not likely influence the results as no temporal trends pertaining to claims associated with OP cancer incidence were anticipated.) A total of 368,666 controls were at least 66 years of age on this pseudo-diagnosis data. Utilizing the same inclusion/exclusion criteria for these subjects for Medicare and no HMO coverage on the pseudo-diagnosis date and its corresponding time window left 242,654 non-OP cancer control subjects.

### Study measures

OP cancers were identified using the SITE RECODE variable from the PEDSF files. Integer values of 1–10 signified the following cancer sites (respectively): lip; tongue; salivary gland; floor of mouth; gum and other mouth; nasopharynx; tonsil; oropharynx; hypopharynx; and other oral cavity and pharynx. For subjects with more than one primary diagnosis at age 66 or older of OP cancer, the occurrence diagnosed in the 2002 calendar year was used. The representative sample of individuals that served as control subjects consisted of observations from the SumDenom file. No variables were needed to identify them as controls, as their position in this 5% sample file identified them as being a Medicare beneficiary living in a SEER area that had not been diagnosed with cancer.

Evaluation of the performance of the algorithm across various demographic characteristics was conducted in the validation process. The following measures contained in both PEDSF and SumDenom files were used for this analysis: age group, sex, and race and ethnicity. Diagnosis and procedure codes from Medicare claims were used for the algorithm to predict whether an individual was an incident OP cancer case or not (a control). The dates that corresponded with these diagnoses and procedures were also located in the Medicare claims, and were used to limit the occurrence of such codes to within one year (before or after) the potential date of incidence.

### Building the algorithm

To generate our algorithm, 1,807 incident OP cancer cases with a date of diagnosis in the 2002 calendar year that met our inclusion criteria were identified. A total of 242,654 subjects from the SumDenom files that were alive in 2002 and randomly assigned a “pseudo-diagnosis date” in the 2002 calendar year. Medicare (MedPAR, NCH, and OutSAF) claims for these subjects that had a date within one year (one year before through one year after) their diagnosis date/pseudo-diagnosis date were selected. ICD-9 procedure codes and CPT procedure codes from these claims were utilized. A 60% simple random sample (without replacement) from these subjects was selected to derive the algorithm (n=1,085 OP cancer cases; n=145,548 controls), leaving the remaining 40% (n=722 OP cancer cases; n=97,106 controls) available for validation. Contingency tables were generated to compare demographic and clinical characteristics of the algorithm building (60%) and validation (40%) samples. These characteristics were compared between samples using Pearson’s chi-square test.

Medicare claims were used to generate weights for the algorithm. Each claim source (MedPAR, NCH, and OutSAF) was treated separately in the process that follows. The first step in the algorithm was to reduce the number of claim types. Thus, using the OP cases, only procedures that had an ICD-9 diagnosis code for OP cancer (values 140.XX-149.XX) at least 50% of the time that the procedure occurred were retained. Next, the relative frequencies of occurrence of (at least one) of each of these unique ICD-9 procedure and CPT codes in the claims among the OP cases were derived, and then again among the controls. The log_2_ of the ratio of these relative frequencies (of presence for each ICD-9 procedure code and each CPT code within each claim source) among the OP cases and among the controls was used to generate a weight for each code. (For codes that occurred only among the OP cases, the relative frequency value used for the control subjects was one divided by the number of controls plus one to avoid division by zero.) Weights with a value less than or equal to four in the ICD-9 procedure codes and CPT codes were set equal to zero. (This was justified on the basis of low discrimination between the relative frequencies among the OP cases versus the controls.) A score for each OP case and for each control was generated by summing the weights for each code that was present during their two-year window around their diagnosis/pseudo-diagnosis dates. Weights for the presence of an ICD-9 diagnosis code value of 140.XX-149.XX (OP cancer diagnosis code) were also generated by taking the log_2_ of the ratio of relative frequencies of occurrence of such a code (in each data source) among the OP cases relative to the non-cancer controls, then adding this weight to each subject’s score. Formally, the equation for determining each subjects score was

(1) $$\begin{array}{rl} \;\text{Score} & =\left({\sum x}_{\text{Med}}\cdot \text{Code}s_{\text{Med}}\right)\\ & \;+\left({\sum x}_{\text{NCH}}\cdot \text{Code}s_{\text{NCH}}\right)\\ & \;+\left({\sum x}_{\text{Out}}\cdot \text{Code}s_{\text{Out}}\right) \end{array}$$

where: *Codes*_*Med*_ represented the various weights from the ICD-9 procedure and diagnosis codes and CPT procedure codes defined above from the MedPAR data source and *x*_*Med*_ represented the corresponding indicator variables (1 if present; 0 if not present) for whether the subject had the code in their claims during the defined time window; *Codes*_*NCH*_ and *x*_*NCH*_ represented these values for the NCH data source; and *Codes*_*Out*_ and *x*_*Out*_ represented them for the OutSAF data source.

Given the scores derived for each data source (MedPAR, NCH, and OutSAF), the algorithm identified subjects as cases if they had a positive value in any one of these three scores. Subjects with a value of zero in all three of these scores were identified as not having OP cancer by the algorithm. Histograms of the scores for each data source and the combined source were presented for OP cancer cases and controls. (Due to the size of the data source for the controls, a simple random sample [without replacement] of those not having OP cancer was used to select subjects for these histograms.) Additional cut-points were also explored and a receiver operating characteristics (ROC) curve [13] was presented. The additional cut-points included the minimum Euclidean distance from the point (0%, 100%) on the ROC curve (representing 100% sensitivity and 100% specificity), and one that maximized specificity. Estimates for sensitivity and specificity, along with their corresponding 95% confidence intervals [13] were generated for each of these cut-points.

### Validating the algorithm

Using the weights for each code used to derive the algorithm, scores were generated for all of the Medicare claims (MedPAR, NCH, and OutSAF) for each subject during their individual time windows (centered on their diagnosis date/pseudo diagnosis date) in the remaining 40% sample, called the validation sample. Subjects that had a positive value in any one of these three scores were identified by the algorithm as having OP cancer, and those with a value of zero in all three of these scores were identified as not having OP cancer. For comparison, the algorithm was also evaluated at the additional cut-points described above. The *a priori* research hypothesis was that the algorithm derived would have sensitivity and specificity values of at least 85% and 95%, respectively. Point estimates and 95% confidence intervals for these sensitivity and specificity [13] were also generated.

Variation in sensitivity and specificity by demographic factors was also evaluated. Using the validation sample, unconditional logistic regression models [14] predicting cancer status as determined by the algorithm were generated, first among those with OP cancer (for sensitivity) and then among the controls (for specificity). Backwards elimination was used to select the model using age group, sex, and race and ethnicity. All possible interactions were allowed, and the selection criterion was set to p<0.1 (Wald test) to remain in the model. The *a priori* research hypothesis was that the sensitivity and specificity values would not vary by demographic subgroups. Predicted probabilities and corresponding 95% confidence intervals were estimated [14]. Model fit was evaluated using the Hosmer-Lemeshow goodness-of-fit test [14].

Positive and negative predictive values were also estimated for the algorithm on the validation sample. These values represented: the probability that a subject identified by the algorithm as an OP cancer case was, in fact, an OP cancer case (positive predictive value); and the probability that a subject indicated by the algorithm as a control subject truly did not have OP cancer (negative predictive value). Because the controls represented only a 5% sample from that population, subjects from this sample were weighted by a factor of 20 to obtain an appropriate estimate for these values. Ninety five percent confidence intervals [13] were estimated for this inflated sample for these estimates.

This project was approved by The University of Kansas Medical Center Human Subjects Committee (HSC #10914). SAS versions 9.1, 9.2, and 9.3 (SAS Institute, Cary, NC) were used for data management and analyses.

## Results

Among the OP cancer cases, the distributions of age group, sex, and race and ethnicity were similar between the algorithm building and validation samples. The same was true for the control samples as well. These results were presented in Table 1.

**Table 1:** Frequency distributions (%) of the characteristics of the algorithm building and validation samples

**Characteristic**		**Algorithm building sample**	**Validation sample**	**Pearson’s*x***^***2***^**test p-value**
OP cancer cases*		1,085 (100.0)	722 (100.0)	
Age Group	66-69	201 (18.5)	116 (16.1)	0.1370
	70-74	271 (25.0)	184 (25.5)	
	75-79	266 (24.5)	195 (27.0)	
	80-84	205 (18.9)	114 (15.8)	
	85 and older	142 (13.1)	113 (15.7)	
Sex	Female	417 (38.4)	287 (39.8)	0.5738
	Male	668 (61.6)	435 (60.3)	
Race and ethnicity	Black	59 (5.4)	43 (6.0)	0.8859
	Hispanic	17 (1.6)	9 (1.3)	
	Other	49 (4.5)	30 (4.2)	
	White	960 (88.5)	640 (88.6)	
**Non-cancer controls**		145,548 (100.0)	97,106 (100.0)	
Age Group	66-69	28,549 (19.6)	19,036 (19.6)	0.2610
	70-74	36,729 (25.2)	24,269 (25.0)	
	75-79	33,361 (22.9)	22,597 (23.3)	
	80-84	24,635 (16.9)	16,479 (17.0)	
	85 and older	22,274 (15.3)	14,725 (15.2)	
Sex	Female	89,736 (61.7)	60,050 (61.8)	0.3564
	Male	55,812 (38.4)	37,056 (38.2)	
Race and ethnicity	Black	10,385 (7.1)	7,148 (7.4)	0.0037
	Hispanic	3,685 (2.5)	2,279 (2.4)	
	Other	8,681 (6.0)	5,897 (6.1)	
	White	122,797 (84.4)	81,782 (84.2)	

*OP: oral and pharyngeal.

### Algorithm built

**Figure 1:**
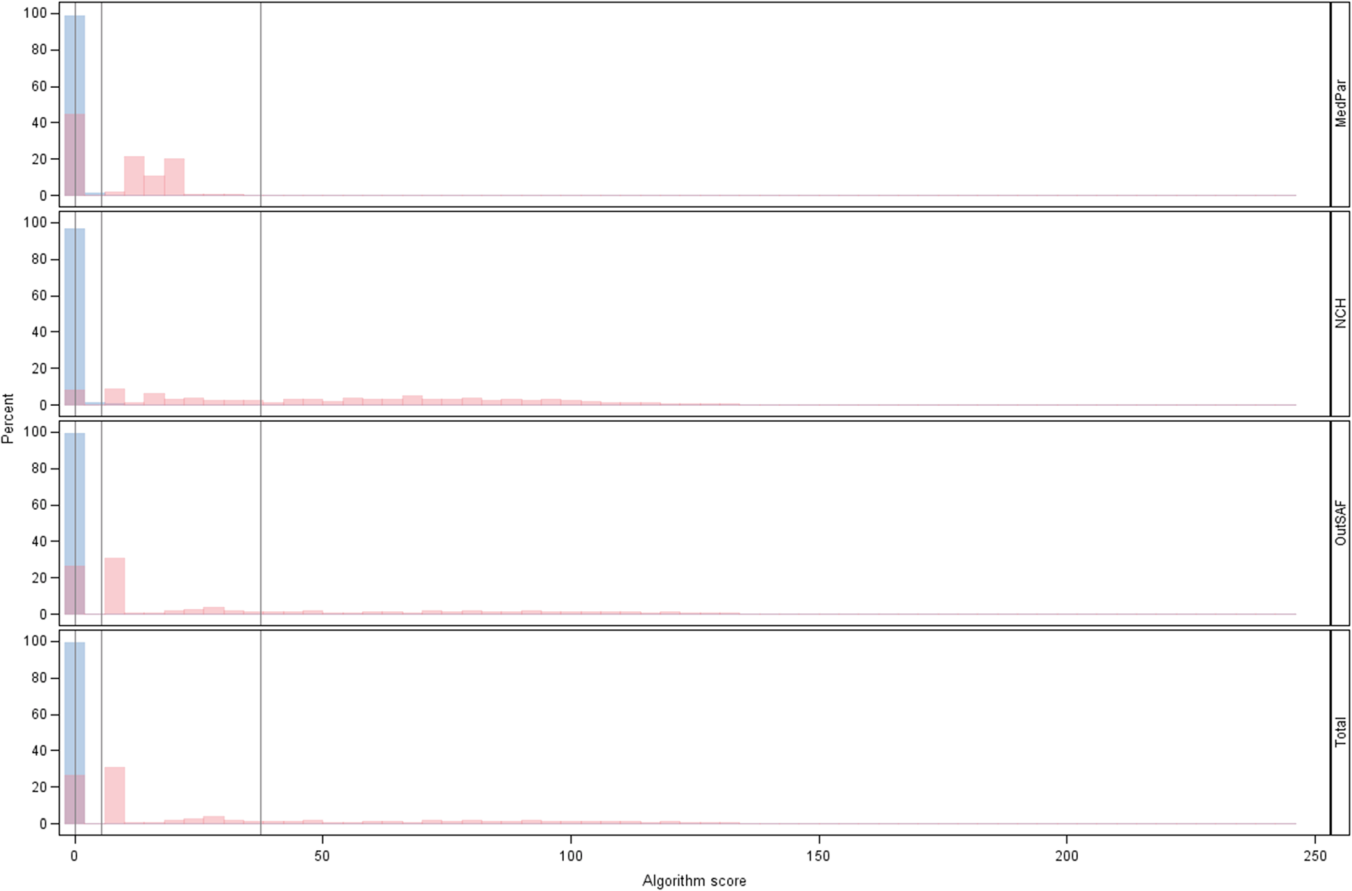
Histograms of the scores based on Medicare claims source and their combined total*. *Oral and pharyngeal (OP) cancer case (magenta) and control (blue) scores; vertical reference bars for: the initial cut-point score (>0 indicating the algorithm identifying as an OP cancer case) that had a sensitivity of 93.9% and specificity of 96.2%, the minimal Euclidean distance cut-point (>5.48) that had a sensitivity of 93.8% and specificity of 97.1%, and for the cut-point that maximized specificity (>37.43) that had 75.0% sensitivity and 99.3% specificity.

**Figure 2:**
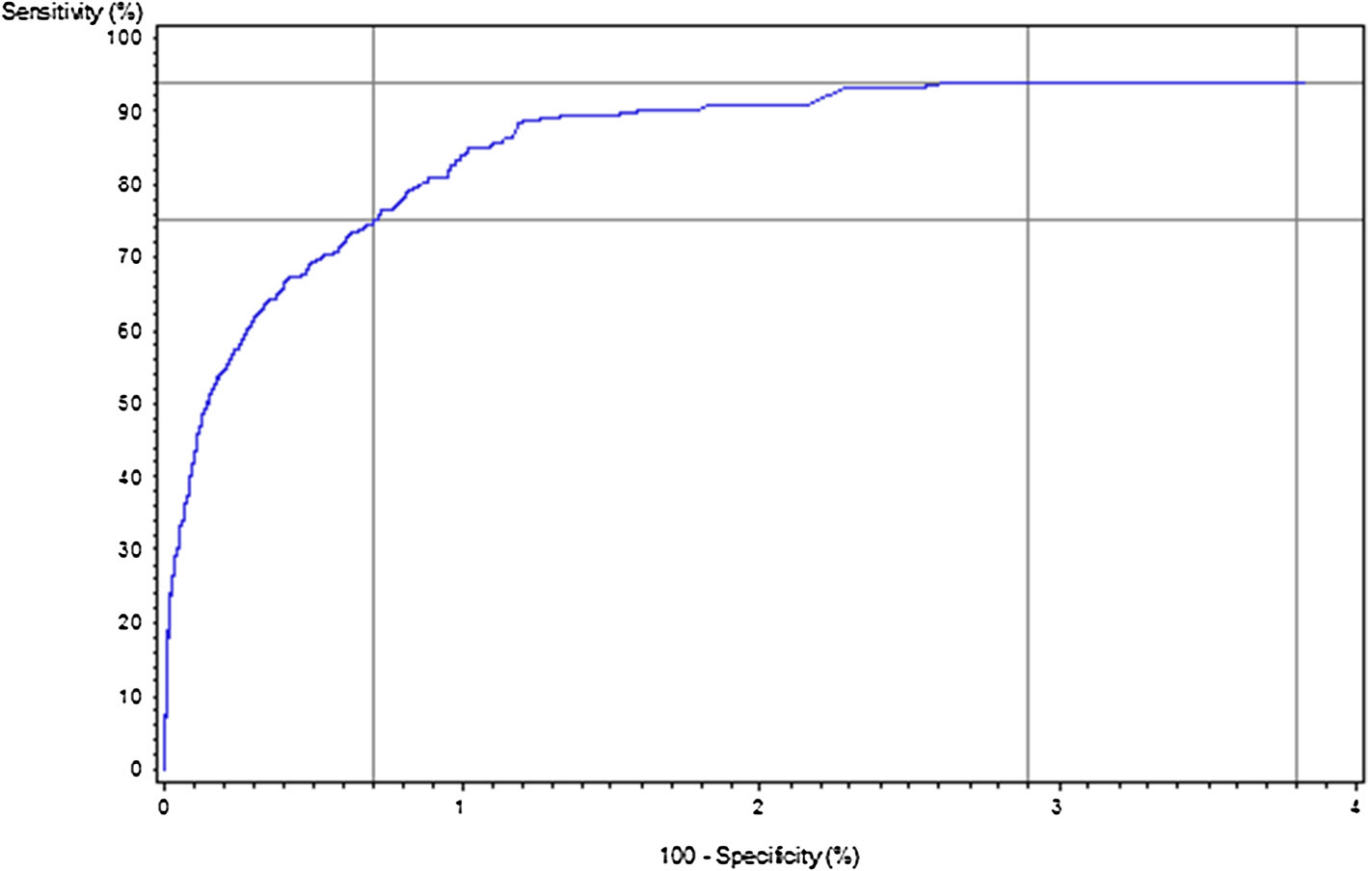
Receiver operating characteristics (ROC) curve for scores based on Medicare claims for identifying incident oral and pharyngeal cancer cases*. *Reference lines indicated: for the initial cut-point score (>0 indicating the algorithm identifying as an OP cancer case) had a sensitivity of 93.9% and specificity of 96.2%; the sensitivity and specificity for the minimum Euclidean distance cut-point (>5.48) were 93.8% and 97.1%, respectively; and for the cut-point that maximized specificity (>37.43), sensitivity was 75.0% and specificity was 99.3%.

The weights of each of the codes to generate overall scores were presented (see Additional file 1: Appendix Tables A-D). Using the cut-point of a positive (>0) score, the sensitivity and specificity were 93.9% and 96.2%, respectively. Histograms of the distributions of scores among the OP cancer cases and controls were presented in Figure 1. The ROC curve for various cut-points of the algorithm score for indicating an OP cancer case was presented in Figure 2, focusing only on values with high specificity (>96%). This figure indicated that the value with the smallest Euclidean distance from the point (0%, 100%) on the ROC curve had high specificity. This minimum distance occurred where the Medicare claims score had a value >5.48, and produced a sensitivity of 93.8% and a 97.1% specificity. Using a more ad hoc approach of prioritizing the maximization of specificity (due to the relatively rare incidence of OP cancer), we found that a cut-point of >37.43 had a sensitivity of 75.0% and a specificity of 99.3%. These results, along with the corresponding 95% confidence intervals, were presented in Table 2.

**Table 2:** Sensitivity and specificity values for various score cut-points for the model building and validation samples

**Medicare claims score cut-point**	Sensitivity (95% CI)*	Specificity (95% CI)*
>0.00	93.9 (92.5-95.3) / 95.3 (93.8-96.8)	96.2 (96.1-96.3) / 96.0 (95.9-96.2)
>5.48	93.8 (92.4-95.3) / 95.3 (93.8-96.8)	97.1 (97.0-97.2) / 97.0 (96.9-97.1)
>37.43	75.0 (72.5-77.6) / 79.8 (76.9-82.7)	99.3 (99.3-99.3) / 99.3 (99.2-99.3)

*%; CI: confidence interval; algorithm building sample values/validation sample values.

### Validation of the algorithm

The various cut-point scores produced similar sensitivity and specificity values to that of the algorithm building sample. The initial cut-point score (>0 indicating the algorithm identifying as an OP cancer case) had a sensitivity of 95.3% and specificity of 96.0%. The sensitivity and specificity for the minimum Euclidean distance cut-point (>5.48) were 95.3% and 97.0%, respectively. For the cut-point that maximized specificity (>37.43), sensitivity was 79.8% and specificity was 99.3%. These results, along with the corresponding 95% confidence intervals, were presented in Table 2.

The model for sensitivity indicated that the sensitivity was lower for males than for females (p=0.0531). The estimated sensitivity was 97.2% for females and 94.2% for males. (Given the number of parameters in this model, the Hosmer-Lemeshow goodness-of-fit test could not be conducted.) The model for specificity was more complex. The final model included age group (p<0.0001), sex (p<0.0001), race and ethnicity (p=0.0158), and the age group-by-race and ethnicity interaction (p=0.0072). No significant lack-of-fit was detected for this model (p=0.5155). All but one age group-by-sex-by-race and ethnicity subgroup had an estimated specificity values exceeding 95%. The group with specificity below this threshold was Hispanic males ages 85 and older, and had a value of 94.0%. The estimated sensitivity and specificity values and corresponding 95% confidence intervals from these models were presented in Table 3. Notably, while statistically significant variations were detected, sensitivity and specificity values were similar across groups.

**Table 3:** Adjusted sensitivity and specificity values for the minimum Euclidean distance cut- point for the validation samples

**Sensitivity (95% confidence interval)**			
Females (regardless of age group and race and ethnicity)			97.2 (94.5-98.6)
Males (regardless of age group and race and ethnicity)			94.0 (91.4-95.9)
**Specificity (95% confidence interval)**			
Ages 66-69	Female	Black	97.3 (96.4-97.9)
		Hispanic	98.0 (96.1-99.0)
		Other	98.2 (97.3-98.7)
		White	97.6 (97.4-97.8)
	Male	Black	96.4 (95.3-97.2)
		Hispanic	97.4 (94.9-98.7)
		Other	97.5 (96.4-98.3)
		White	96.8 (96.5-97.2)
Ages 70-74	Female	Black	97.6 (96.8-98.2)
		Hispanic	98.3 (97.2-99.0)
		Other	98.2 (97.5-98.8)
		White	97.1 (96.9-97.4)
	Male	Black	96.8 (95.8-97.6)
		Hispanic	97.8 (96.3-98.7)
		Other	97.7 (96.7-98.3)
		White	96.2 (95.9-98.5)
Ages 75-79	Female	Black	97.5 (96.6-98.1)
		Hispanic	98.2 (97.0-99.0)
		Other	98.1 (97.3-98.7)
		White	97.0 (96.7-97.2)
	Male	Black	95.0 (93.7-96.1)
		Hispanic	96.8 (95.0-98.0)
		Other	97.3 (96.2-98.1)
		White	96.0 (95.6-96.3)
Ages 80-84	Female	Black	97.2 (96.1-98.0)
		Hispanic	96.6 (94.2-98.0)
		Other	96.8 (95.5-97.7)
		White	97.2 (96.9-97.4)
	Male	Black	96.3 (94.8-97.4)
		Hispanic	95.5 (92.4-97.4)
		Other	95.7 (94.0-97.0)
		White	96.3 (95.9-96.6)
Ages 85 and older	Female	Black	96.7 (95.4-97.6)
		Hispanic	95.4 (91.7-97.5)
		Other	97.4 (96.0-98.3)
		White	97.8 (97.5-98.0)
	Male	Black	95.6 (93.9-96.8)
		Hispanic	94.0 (89.1-96.7)
		Other	96.5 (94.7-97.7)
		White	97.1 (96.7-97.4)

The positive predictive values for each of these algorithm cut-points were very low; although with the large sample sizes the confidence intervals were narrow. The initial cut-point score (>0 indicating the algorithm identifying as an OP cancer case) had a value of 0.9% (0.8-1.0%). For the minimum Euclidean distance cut-point (>5.48) the positive predictive value was 1.2% (1.1-1.2%). For the cut-point that maximized specificity (>37.43), the positive predictive value was 4.0% (3.6-4.3%). The negative predictive values, conversely, were extremely high. For the initial and Euclidean distance cut-points, these values were 100.0% (<100.0-100.0%). For the maximized specificity cut-point, the negative predictive value was 99.9% (99.9-99.9%).

## Discussion

A new algorithm based on Medicare claims to identify incident OP cancer cases was created. This algorithm used the relative frequency of occurrence of various diagnosis and procedure codes to determine weights. By summing these weights when present in individuals’ claims, scores were derived such that a higher score was indicative of the algorithm identifying subjects as incident OP cancer cases. The algorithm was based upon an empirical process that identified and scored various claims from three different Medicare sources. Measures of sensitivity and specificity exceeded *a priori* threshold values (85% and 95%, respectively). Notably, the use of only a subset of the three Medicare claims sources would negatively impact the sensitivity by possibly missing claims that would increase a score to indicate OP cancer, whereas this would have a positive impact on specificity by not adding to the score a claim from that source among subjects without OP cancer.

The algorithm identified subjects as OP cancer cases based on passing a score threshold based on their Medicare claims. The impact of the removal of one or more of the claims sources (MedPAR, NCH, and/or OutSAF) would, therefore, modify (reduce) the sensitivity of the algorithm by reducing the number of subjects identified as cases. In contrast, the specificity would be increased as this would reduce the potential of the algorithm miss-identifying a control subject as an OP cancer case.

The utility of this algorithm depends on several factors. For example, in trying to examine health trajectories over time using Medicare claims, algorithms with a low positive predictive value may be of little use. This follows from the fact that more subjects identified by the algorithm as cases will actually be free of disease when the positive predictive value is less than 50%. This is true even for high values of sensitivity and specificity, as found in the algorithm derived in this study. In comparison to other algorithms, Freeman et al. [6] were able to identify an algorithm cut-point for breast cancer that achieved high sensitivity (90%) and specificity (>99%), and a positive predictive value exceeding 70%. The algorithm by Nattinger et al. [10], also for breast cancer, increased the positive predictive value to nearly 90%. Low positive predictive values are a concern in situations, such as for the algorithm derived in this study, where the underlying disease is rare as near 100% specificity is required to have a high positive predictive value. The extremely high negative predictive value, conversely, could make this a useful tool for a “rule out” decision when the algorithm indicates the subject is OP cancer free (e.g., for a registry trying to reduce the number of potential cases it needs to follow-up).

In terms of estimating baseline and trend data over time, this algorithm could be used despite the low positive predictive value. For example, one could obtain Medicare claims for regions not covered by a registry, or a registry could use this algorithm as a point of comparison in evaluating their completeness. Using Medicare claims, the algorithm would indicate which subjects were indicated as cases and which were not. The estimated positive predictive value (or its confidence interval boundaries) could be multiplied by the number of algorithm-positive cases. Similarly, the number of algorithm-negative subjects could be multiplied by 100% minus the negative predictive value (or its confidence interval boundaries) to estimate the number of cases missed by the algorithm. Summing the positive predictive value-corrected number of algorithm-positive subjects with the negative predictive value-corrected estimate of the number of cases missed by the algorithm would provide the estimated number of subjects with OP cancer. In addition to baseline and trend data monitoring, this approach could also be used for generating data for ecologic level analyses.

### Limitations

The algorithm derived for this study used Medicare beneficiaries diagnosed with OP cancer in 2002 and subjects from a SEER area that did not link to the SEER tumor registry. As noted previously, subjects with cancers other than OP cancer were not included in these analyses. Consequently, the specificity (and thus positive and negative predictive values) was (were) affected by their exclusion. However, SEER estimated the (age-adjusted) incidence of cancer in 2008 from all sites among those ages 65 years and older at approximately 2,072/100,000 person-years [15]. Thus, the worst case scenario is that the specificity would have been approximately 1-2% lower; but most likely the impact on specificity would have been even less. Also pertaining to subjects, this algorithm was derived among subjects at least 66 years of age with Medicare coverage (and no HMO coverage) during the time-window around the diagnosis (and pseudo-diagnosis) dates as described above. Thus, operating characteristics may be different among subjects not meeting these criteria if such characteristics would alter the sensitivity and/or specificity in a systematic way. However, our results that assessed for variation in sensitivity and specificity failed to detect age variation in sensitivity, and while statistically significant, the variations in specificity were minimal (Table 3).

Another limitation of this study was that the codes may change over time. For example, entire coding systems may be upgraded over time (e.g., shifting from ICD-9 to ICD-10). Thus, use of such an algorithm for baseline and trend estimates of OP cancer incidence among those 66 years of age and older may need periodic refinement using the methods described above. The population of interest may have different demographic characteristics than those used to derive these measures. To account for this, estimates could be done within various age group/sex/race and ethnicity categories as needed using values such as those presented in Table 3. While refinement can update the algorithm with respect to its ability to identify OP cancer cases within this large population of Medicare beneficiaries, the utility of the weights to derive scores for subjects in other important populations, such as those less than 66 years of age, could not be evaluated with the data used for this study. However, the approached described could be applied to other data sources of similar structure, such as a large health insurance claims source of hospital and clinic electronic medical records.

It is noteworthy that the sensitivity and specificity of the algorithm were driven in part by the length of time set to look for codes within the Medicare claims. A longer window would increase the ability to capture more codes—thereby allowing for sensitivity to be increased, while a shorter window would conversely benefit specificity. Further, the approach does not provide an exact date of diagnosis; it does, however, provide for an estimated window based on the duration of time used to look for the various claims.

## Conclusions

Medicare claims data were used create a new algorithm to identify incident cases of OP cancer. Though the sensitivity and specificity for this algorithm were high, the positive predictive value was low. Examples of the utility of this algorithm and its operating characteristics include using it to derive baseline and trend estimates of OP cancer incidence. Such measures could be used to provide incidence estimates where they are lacking or to serve as comparator estimates for tumor registries.

## Competing interests

The authors declare that they have no competing interests.

## Authors’ contributions

JDM, DG, and MM participating in the design of this study. JDM and JK conducted the statistical analyses. XC determined code categorizations. JDM wrote the manuscript, and all authors participated in the review and editing for intellectual content. All authors have read and approved the final manuscript.

## Pre-publication history

The pre-publication history for this paper can be accessed here:

http://www.biomedcentral.com/1472-6831/13/1/prepub

## Supplementary Material

Additional file 1 **Appendix A.** Appendix Table A Score weight from MedPAR ICD-9-CM procedure (diagnosis) codes. Table B Score weight from NCH CPT procedure (ICD-9-CM diagnosis) codes. Table C Score weight from OutSAF ICD-9-CM and CPT procedure (ICD-9-CM diagnosis) codes*. Table D Score weight from MedPAR, NCH, and OutSAF ICD-9-CM and CPT procedure (ICD-9-CM diagnosis) codes by clinical categories*. Click here for file
