# Supplementary material for: Identifying incident oral and pharyngeal cancer cases using Medicare claims
Source: BMC Oral Health. 2013 Jan 1;13:1. doi: 10.1186/1472-6831-13-1 (PMC3538504; doi:10.1186/1472-6831-13-1)
Supplement: Additional file 1 — Appendix A. Appendix Table A Score weight from MedPAR ICD-9-CM procedure (diagnosis) codes. Table B Score weight from NCH CPT procedure (ICD-9-CM diagnosis) codes. Table C Score weight from OutSAF ICD-9-CM and CPT procedure (ICD-9-CM diagnosis) codes*. Table D Score weight from MedPAR, NCH, and OutSAF ICD-9-CM and CPT procedure (ICD-9-CM diagnosis) codes by clinical categories*. [file 1472-6831-13-1-S1.docx]

Appendix A. Appendix Table A Score weight from MedPAR ICD-9-CM procedure (diagnosis) codes

| Procedure codes | Weight |
| --- | --- |
| 140.XX-149.XX (diagnosis codes) | 10.3869 |
| 33.23 | 4.0318 |
| 22.2 | 4.0677 |
| 40.19 | 4.0677 |
| 92.27 | 4.1488 |
| 99.28 | 4.1608 |
| 18.79 | 4.2603 |
| 26.0 | 4.2603 |
| 77.77 | 4.2603 |
| 87.39 | 4.2603 |
| 97.41 | 4.2603 |
| 02.94 | 4.3128 |
| 40.3 | 4.3163 |
| 46.39 | 4.3373 |
| 31.74 | 4.3672 |
| 97.51 | 4.4432 |
| 31.44 | 4.4827 |
| 58.22 | 4.4827 |
| 97.39 | 4.4827 |
| 99.08 | 4.4827 |
| 99.74 | 4.6753 |
| 21.09 | 4.7457 |
| 22.62 | 4.7457 |
| 27 69 | 4.7457 |
| 86.67 | 4.8453 |
| 33.21 | 4.8977 |
| 86.69 | 4.9133 |
| 43.19 | 5.0421 |
| 86.72 | 5.0677 |
| 06.02 | 5.0677 |
| 18.6 | 5.0677 |
| 21.69 | 5.0677 |
| 31.69 | 5.0677 |
| 42.41 | 5.0677 |
| 52.14 | 5.0677 |
| 77.31 | 5.0677 |
| 83.43 | 5.0677 |
| 83.87 | 5.0677 |
| 87.43 | 5.0677 |
| 97.31 | 5.0677 |
| 83.21 | 5.3021 |
| 31.1 | 5.3683 |
| 29.11 | 5.4302 |
| 31.5 | 5.4827 |
| 34.3 | 5.4827 |
| 39.91 | 5.4827 |
| 40.59 | 5.4827 |
| 43.0 | 5.4827 |
| 44.63 | 5.4827 |
| 76.93 | 5.4827 |
| 20.01 | 5.6526 |
| 83.82 | 5.6691 |
| 29.4 | 5.7457 |
| 83.32 | 5.7457 |
| 97.23 | 5.7651 |
| 86.74 | 5.7930 |
| 27.0 | 5.8977 |
| 86.70 | 5.9302 |
| 42.24 | 5.9522 |
| 31.29 | 5.9828 |
| 06.09 | 6.0677 |
| 06.12 | 6.0677 |
| 08.89 | 6.0677 |
| 21.22 | 6.0677 |
| 31.41 | 6.0677 |
| 31.45 | 6.0677 |
| 42.81 | 6.0677 |
| 84.05 | 6.0677 |
| 92.22 | 6.0677 |
| 92.23 | 6.0677 |
| 20.49 | 6.3307 |
| 38.82 | 6.3307 |
| 76.76 | 6.3307 |
| 99.25 | 6.4011 |
| 06.31 | 6.4827 |
| 86.71 | 6.4827 |
| 31.42 | 6.5882 |
| 23.09 | 6.6082 |
| 04.07 | 6.7457 |
| 08.59 | 6.8046 |
| 23.19 | 6.8612 |
| 92.24 | 6.9302 |
| 92.29 | 7.0412 |
| 06.91 | 7.0677 |
| 08.36 | 7.0677 |
| 08.70 | 7.0677 |
| 08.99 | 7.0677 |
| 18.09 | 7.0677 |
| 22.02 | 7.0677 |
| 23.6 | 7.0677 |
| 24.12 | 7.0677 |
| 24.4 | 7.0677 |
| 24.91 | 7.0677 |
| 25.51 | 7.0677 |
| 25.94 | 7.0677 |
| 26.91 | 7.0677 |
| 27.23 | 7.0677 |
| 27.29 | 7.0677 |
| 27.99 | 7.0677 |
| 28.5 | 7.0677 |
| 28.99 | 7.0677 |
| 29.2 | 7.0677 |
| 42.11 | 7.0677 |
| 42.22 | 7.0677 |
| 42.25 | 7.0677 |
| 76.11 | 7.0677 |
| 76.45 | 7.0677 |
| 76.46 | 7.0677 |
| 76.64 | 7.0677 |
| 77.73 | 7.0677 |
| 86.81 | 7.0677 |
| 97.01 | 7.0677 |
| 07.62 | 7.3572 |
| 24.5 | 7.3896 |
| 40.21 | 7.3896 |
| 76.92 | 7.3896 |
| 24.11 | 7.4827 |
| 26.31 | 7.5333 |
| 29.59 | 7.6526 |
| 30.3 | 7.6526 |
| 31.43 | 7.6843 |
| 26.29 | 7.8046 |
| 30.29 | 7.8046 |
| 22.11 | 8.0677 |
| 27.72 | 8.0677 |
| 29.53 | 8.0677 |
| 31.95 | 8.0677 |
| 76.41 | 8.0677 |
| 26.32 | 8.3156 |
| 76.2 | 8.5271 |
| 24.7 | 8.6526 |
| 25.3 | 8.6526 |
| 26.49 | 8.6526 |
| 27.22 | 8.6526 |
| 28.11 | 8.6526 |
| 28.92 | 8.6526 |
| 76.91 | 8.6526 |
| 27.32 | 8.8750 |
| 27.56 | 8.8750 |
| 25.02 | 8.9421 |
| 24.31 | 9.0677 |
| 26.12 | 9.0677 |
| 26.30 | 9.0677 |
| 27.31 | 9.0677 |
| 27.42 | 9.0677 |
| 31.75 | 9.0677 |
| 28.2 | 9.2376 |
| 76.43 | 9.2900 |
| 25.59 | 9.3896 |
| 27.55 | 9.3896 |
| 29.39 | 9.3896 |
| 25.1 | 9.5912 |
| 04.5 | 9.6526 |
| 08.52 | 9.6526 |
| 25.4 | 9.6526 |
| 27.24 | 9.6526 |
| 27.57 | 9.7681 |
| 40.40 | 9.8750 |
| 29.12 | 10.1033 |
| 40.41 | 10.1294 |
| 27.49 | 10.1762 |
| 25.01 | 10.1831 |
| 76.39 | 10.3896 |
| 27.59 | 10.5271 |
| 76.31 | 10.5271 |
| 40.42 | 10.6526 |
| 29.33 | 10.7681 |
| 25.2 | 11.3751 |
| 30.4 | 11.4600 |

**Appendix Table B** Score weight from NCH CPT procedure (ICD-9-CM diagnosis) codes

| Procedure codes | Weight |
| --- | --- |
| 140.XX-149.XX (diagnosis codes) | 8.2513 |
| 43219 | 4.0675 |
| 21110 | 4.2601 |
| 31588 | 4.2601 |
| 77776 | 4.2601 |
| 77782 | 4.2601 |
| 77782 | 4.2601 |
| 78201 | 4.2601 |
| 95933 | 4.2601 |
| 31613 | 4.4825 |
| 76400 | 4.4825 |
| 35261 | 4.7455 |
| 31530 | 5.0675 |
| 32525 | 5.0675 |
| 38305 | 5.0675 |
| 41250 | 5.0675 |
| 44201 | 5.0675 |
| 77761 | 5.0675 |
| 21032 | 5.4825 |
| 31599 | 5.4825 |
| 42160 | 5.4825 |
| 61580 | 5.4825 |
| 69535 | 5.4825 |
| 37605 | 6.0675 |
| 4100 | 6.0675 |
| 42660 | 6.0675 |
| 07740 | 7.0675 |
| 12057 | 7.0675 |
| 20970 | 7.0675 |
| 21070 | 7.0675 |
| 21193 | 7.0675 |
| 21196 | 7.0675 |
| 31367 | 7.0675 |
| 31368 | 7.0675 |
| 31370 | 7.0675 |
| 31560 | 7.0675 |
| 37565 | 7.0675 |
| 40842 | 7.0675 |
| 40899 | 7.0675 |
| 41140 | 7.0675 |
| 41252 | 7.0675 |
| 42182 | 7.0675 |
| 42325 | 7.0675 |
| 61576 | 7.0675 |
| 61590 | 7.0675 |
| 64740 | 7.0675 |
| 64742 | 7.0675 |
| 64902 | 7.0675 |
| 67902 | 7.0675 |
| 69720 | 7.0675 |
| 88239 | 7.0675 |
| 15200 | 4.2601 |
| 44130 | 4.6080 |
| 61626 | 4.6080 |
| 15620 | 4.8975 |
| 35701 | 4.8975 |
| 77408 | 4.8975 |
| 43653 | 5.2601 |
| 15756 | 5.7455 |
| 21249 | 6.0675 |
| 42953 | 6.0675 |
| 15840 | 6.4825 |
| 37600 | 6.4825 |
| 42299 | 6.4825 |
| 42500 | 6.4825 |
| 42450 | 7.0675 |
| 42505 | 7.0675 |
| 69502 | 7.0675 |
| 20955 | 8.0675 |
| 31390 | 8.0675 |
| 42107 | 8.0675 |
| 42200 | 8.0675 |
| 42845 | 8.0675 |
| 4299 | 8.0675 |
| 43300 | 8.0675 |
| 43496 | 8.0675 |
| 64716 | 8.0675 |
| 64885 | 8.0675 |
| 60200 | 4.8451 |
| 31540 | 4.9520 |
| 41825 | 5.3305 |
| 35201 | 5.8451 |
| 21215 | 6.6524 |
| 77777 | 7.0675 |
| 21082 | 8.6524 |
| 31395 | 8.6524 |
| 40527 | 8.6524 |
| 40845 | 8.6524 |
| 41153 | 8.6524 |
| 42699 | 8.6524 |
| 64886 | 8.6524 |
| 42106 | 4.9800 |
| 21085 | 5.0675 |
| 21299 | 6.2601 |
| 31576 | 6.2601 |
| 42808 | 6.4825 |
| 38542 | 7.4825 |
| 21081 | 8.0675 |
| 31360 | 8.0675 |
| 42892 | 8.0675 |
| 41145 | 9.0675 |
| 41599 | 9.0675 |
| 42870 | 9.0675 |
| 42894 | 9.0675 |
| 42950 | 9.0675 |
| 77399 | 9.0675 |
| 41826 | 5.5820 |
| 31515 | 5.6889 |
| 40525 | 6.3894 |
| 92502 | 7.8044 |
| 42280 | 8.3894 |
| 21044 | 9.3894 |
| 31365 | 9.3894 |
| 42844 | 9.3894 |
| 43832 | 5.0086 |
| 41857 | 7.0675 |
| 42425 | 8.6524 |
| 41874 | 5.7873 |
| 40500 | 6.5529 |
| 31526 | 7.8748 |
| 21076 | 8.2899 |
| 42426 | 8.2899 |
| 42802 | 8.8748 |
| 15758 | 9.8748 |
| 21080 | 9.8748 |
| 15350 | 4.8975 |
| 42842 | 10.0675 |
| 42890 | 10.0675 |
| 77412 | 6.1499 |
| 20969 | 9.2374 |
| 21198 | 8.3894 |
| 41150 | 10.3894 |
| 15734 | 4.2790 |
| 41899 | 5.0350 |
| 78810 | 5.0675 |
| 40816 | 6.4394 |
| 21089 | 6.8264 |
| 41113 | 6.9419 |
| 77262 | 4.0675 |
| 77416 | 5.5231 |
| 96414 | 5.5584 |
| 42440 | 6.0675 |
| 40520 | 6.1240 |
| 21079 | 9.1829 |
| 15757 | 9.7679 |
| 41130 | 9.8748 |
| 41112 | 5.3596 |
| 40814 | 6.8044 |
| 38700 | 7.5149 |
| 38720 | 8.3894 |
| 21244 | 9.4825 |
| 40812 | 4.7007 |
| 70320 | 6.2279 |
| 77418 | 6.2279 |
| 42420 | 7.5080 |
| 42120 | 8.7304 |
| 42100 | 6.3019 |
| 15732 | 5.3937 |
| 77310 | 6.6018 |
| 41105 | 7.3570 |
| 41116 | 7.4394 |
| 41108 | 7.0675 |
| 77285 | 5.6129 |
| 15120 | 5.0935 |
| 77414 | 5.0675 |
| 41135 | 10.3405 |
| 40808 | 5.5917 |
| 42800 | 7.8975 |
| 77370 | 5.3736 |
| 41155 | 10.3154 |
| 41100 | 6.4708 |
| 77301 | 6.5346 |
| 77333 | 6.5736 |
| 31600 | 4.2443 |
| 70355 | 4.6812 |
| 41120 | 9.1484 |
| 77332 | 5.1772 |
| 00176 | 10.6035 |
| 77305 | 6.7128 |
| 31525 | 5.5139 |
| 96412 | 4.4470 |
| 38724 | 9.1455 |
| 31535 | 7.8107 |
| 96410 | 4.4019 |
| 77413 | 6.0824 |
| 00170 | 5.8830 |
| 76370 | 5.5838 |
| 43200 | 6.9807 |
| 77336 | 5.5183 |
| 77417 | 5.8768 |
| 77321 | 6.7435 |
| 77331 | 5.5913 |
| 77295 | 5.4437 |
| 77470 | 5.4825 |
| 77280 | 5.7547 |
| 77315 | 6.1275 |
| 77290 | 5.7577 |
| 77263 | 5.6411 |
| 77300 | 5.5405 |
| 77334 | 5.8771 |
| 77427 | 5.7690 |

**Appendix Table C** Score weight from OutSAF ICD-9-CM and CPT procedure (ICD-9-CM diagnosis) codes*

| Procedure codes | Weight |
| --- | --- |
| 140.XX-149.XX (diagnosis codes) | 9.4046 |
| 27.23 | 4.0675 |
| 26.0 | 4.2603 |
| 92.19 | 4.4827 |
| 24.31 | 4.7457 |
| 77.41 | 4.7457 |
| 22.11 | 5.0677 |
| 89.31 | 5.0677 |
| 96.08 | 5.0677 |
| 97.39 | 5.0677 |
| 20.09 | 5.4827 |
| 27.72 | 5.4827 |
| 26.91 | 6.0677 |
| 24.12 | 7.0677 |
| 25.09 | 7.0677 |
| 26.49 | 7.0677 |
| 26.99 | 7.0677 |
| 27.21 | 7.0677 |
| 28.0 | 7.0677 |
| 44.39 | 7.0677 |
| 92.12 | 7.0677 |
| 92.23 | 7.0677 |
| 92.25 | 7.0677 |
| 95.43 | 7.0677 |
| 24.5 | 4.3672 |
| 46.32 | 4.3672 |
| 87.39 | 4.3672 |
| 23.09 | 4.4827 |
| 96.05 | 5.0677 |
| 76.2 | 5.4827 |
| 25.59 | 6.4827 |
| 23.19 | 4.0677 |
| 43.19 | 5.3307 |
| 24.4 | 6.3307 |
| 27.31 | 7.0677 |
| 24.11 | 8.6526 |
| 27.32 | 8.6526 |
| 28.92 | 8.6526 |
| 29.39 | 6.2603 |
| 26.12 | 9.0677 |
| 27.22 | 9.0677 |
| 28.2 | 5.8453 |
| 26.31 | 5.9522 |
| 27.56 | 8.2900 |
| 27.59 | 7.4827 |
| 28.11 | 10.2376 |
| 29.11 | 6.7197 |
| 26.32 | 6.8266 |
| 99.25 | 4.4282 |
| 27.57 | 6.5202 |
| 27.42 | 7.0677 |
| 27.24 | 8.5531 |
| 33.22 | 4.3869 |
| 25.1 | 6.7628 |
| 25.2 | 11.2376 |
| 25.02 | 9.4600 |
| 33.23 | 6.2197 |
| 92.24 | 6.4827 |
| 92.29 | 6.4156 |
| 27.49 | 8.2900 |
| 86.07 | 4.4569 |
| 25.01 | 9.2197 |
| 29.12 | 8.2900 |
| 27.43 | 6.7390 |
| 43.11 | 4.7823 |
| 31.43 | 7.2376 |
| 42.23 | 5.5309 |
| 31.42 | 6.5089 |
| 31599 | 4.0677 |
| 69220 | 4.0677 |
| 74355 | 4.0677 |
| 92612 | 4.0677 |
| 15851 | 4.2603 |
| 77783 | 4.2603 |
| 38999 | 4.4827 |
| 87420 | 4.4827 |
| 96520 | 4.4827 |
| D0140 | 4.4827 |
| J9040 | 4.4827 |
| 15630 | 4.7457 |
| J9250 | 4.7457 |
| D7140 | 5.0677 |
| J1110 | 5.0677 |
| 31899 | 5.4827 |
| 41825 | 5.4827 |
| 43219 | 5.4827 |
| 46750 | 5.4827 |
| 77409 | 5.4827 |
| G0251 | 5.4827 |
| J2820 | 5.4827 |
| P9038 | 5.4827 |
| 31830 | 6.0677 |
| 41830 | 6.0677 |
| 62365 | 6.0677 |
| 77407 | 6.0677 |
| G0174 | 6.0677 |
| J1450 | 6.0677 |
| 15770 | 7.0677 |
| 31515 | 7.0677 |
| 40650 | 7.0677 |
| 40652 | 7.0677 |
| 40654 | 7.0677 |
| 40799 | 7.0677 |
| 41827 | 7.0677 |
| 42500 | 7.0677 |
| 42806 | 7.0677 |
| 42870 | 7.0677 |
| 43653 | 7.0677 |
| 57155 | 7.0677 |
| 71551 | 7.0677 |
| 86337 | 7.0677 |
| 92605 | 7.0677 |
| 92609 | 7.0677 |
| A4636 | 7.0677 |
| C1717 | 7.0677 |
| D7111 | 7.0677 |
| D7210 | 7.0677 |
| E0781 | 7.0677 |
| J9600 | 7.0677 |
| 44372 | 4.1608 |
| 44500 | 4.4827 |
| 43830 | 5.0677 |
| 77402 | 5.7457 |
| 41826 | 6.4827 |
| 77327 | 6.4827 |
| 15620 | 7.0677 |
| 42106 | 7.0677 |
| 77263 | 7.0677 |
| 21044 | 8.0677 |
| 40816 | 8.0677 |
| 40820 | 8.0677 |
| 41114 | 8.0677 |
| 42107 | 8.0677 |
| 96530 | 4.7457 |
| 77784 | 5.4827 |
| 42415 | 5.6526 |
| 42410 | 5.8453 |
| 77404 | 6.0677 |
| 42400 | 6.3307 |
| 42104 | 6.6526 |
| 41113 | 7.0677 |
| 40520 | 7.6526 |
| 77427 | 7.6526 |
| 31510 | 8.6526 |
| 41130 | 8.6526 |
| 42802 | 8.6526 |
| G0178 | 8.6526 |
| J9170 | 4.8197 |
| 42440 | 4.8977 |
| 70488 | 5.0677 |
| Q0180 | 5.7457 |
| 42808 | 6.4827 |
| 42120 | 9.0677 |
| 31576 | 6.3896 |
| 41110 | 7.8046 |
| 42804 | 8.3896 |
| 40810 | 9.3896 |
| J9190 | 4.1932 |
| 42826 | 6.1932 |
| 40812 | 7.0677 |
| 42100 | 9.8750 |
| 40500 | 8.0677 |
| 40525 | 8.4827 |
| G0223 | 9.0677 |
| 41112 | 6.6526 |
| 42999 | 7.9157 |
| 41116 | 9.2376 |
| 40808 | 10.2376 |
| 42420 | 8.0677 |
| 42800 | 10.3896 |
| 77403 | 5.4396 |
| 74350 | 6.1348 |
| 77310 | 6.5271 |
| 40510 | 8.9421 |
| J9265 | 5.1932 |
| 77285 | 5.9522 |
| C1788 | 6.0677 |
| J9060 | 6.2445 |
| 40530 | 7.5982 |
| 41108 | 8.7681 |
| 41105 | 9.1831 |
| 77408 | 7.4156 |
| 70355 | 5.3598 |
| 41120 | 10.9745 |
| J9045 | 5.0453 |
| 92511 | 6.5441 |
| 41100 | 9.0677 |
| 88321 | 4.7610 |
| G0224 | 8.7306 |
| J0207 | 7.5271 |
| 77412 | 7.4213 |
| 77333 | 6.3963 |
| 31536 | 6.8225 |
| 77301 | 6.7051 |
| 77416 | 5.8660 |
| Q0084 | 4.6210 |
| 77305 | 7.0219 |
| 77418 | 6.9828 |
| 31525 | 7.5113 |
| 77332 | 5.1857 |
| 77370 | 5.3383 |
| 31622 | 4.5691 |
| 43246 | 4.1651 |
| 31575 | 5.9448 |
| 77470 | 5.3603 |
| 77414 | 4.9659 |
| 77321 | 6.5468 |
| 43200 | 7.4951 |
| 31535 | 8.7636 |
| 77295 | 5.6057 |
| 77331 | 5.7655 |
| 88331 | 4.1654 |
| 76370 | 5.8385 |
| 77315 | 6.1532 |
| 77280 | 5.9866 |
| 77413 | 6.4289 |
| 77290 | 6.0012 |
| 77417 | 5.9818 |
| 77300 | 5.8020 |
| 77336 | 5.8683 |
| 77334 | 6.0157 |

*ICD-9-CM procedure codes of format XX.XX; CPT procedure codes of format XXXXX

**Appendix Table D** Score weight from MedPAR, NCH, and OutSAF ICD-9-CM and CPT procedure (ICD-9-CM diagnosis) codes by clinical categories*

| Description | Data source | Code | Weight |
| --- | --- | --- | --- |
|  | MedPAR | 140.XX-149.XX (diagnosis codes) | 10.3869 |
|  | OutSAF | 140.XX-149.XX (diagnosis codes) | 9.4046 |
|  | NCH | 140.XX-149.XX (diagnosis codes) | 8.2513 |
| Anesthesia\ \Anesthesia | NCH | 00176 | 10.6035 |
|  | NCH | 00170 | 5.8830 |
| Diagnostic Radiology | MedPAR | 87.43 | 5.0677 |
|  | OutSAF | 87.39 | 4.3672 |
|  | MedPAR | 87.39 | 4.2603 |
| Excision of larynx | MedPAR | 30.4 | 11.4600 |
|  | MedPAR | 30.29 | 7.8046 |
|  | MedPAR | 30.3 | 7.6526 |
| Incision and excision of stomach | MedPAR | 43.0 | 5.4827 |
|  | OutSAF | 43.19 | 5.3307 |
|  | MedPAR | 43.19 | 5.0421 |
|  | OutSAF | 43.11 | 4.7823 |
| Incision excision, and occlusion of vessels | MedPAR | 38.82 | 6.3307 |
| Incision, excision, and division of other bones | MedPAR | 77.73 | 7.0677 |
|  | MedPAR | 77.31 | 5.0677 |
|  | OutSAF | 77.41 | 4.7457 |
|  | MedPAR | 77.77 | 4.2603 |
| Interview, evaluation , consultation and examination | OutSAF | 89.31 | 5.0677 |
| Level II HCPCS codes\A codes\ | OutSAF | A4636 | 7.0677 |
| Level II HCPCS codes\C codes\ | OutSAF | C1717 | 7.0677 |
|  | OutSAF | C1788 | 6.0677 |
| Level II HCPCS codes\D codes\ | OutSAF | D7111 | 7.0677 |
|  | OutSAF | D7210 | 7.0677 |
|  | OutSAF | D7140 | 5.0677 |
|  | OutSAF | D0140 | 4.4827 |
| Level II HCPCS codes\E codes\ | OutSAF | E0781 | 7.0677 |
| Level II HCPCS codes\G codes\ | OutSAF | G0223 | 9.0677 |
|  | OutSAF | G0224 | 8.7306 |
|  | OutSAF | G0178 | 8.6526 |
|  | OutSAF | G0174 | 6.0677 |
|  | OutSAF | G0251 | 5.4827 |
| Level II HCPCS codes\J codes\ | OutSAF | J0207 | 7.5271 |
|  | OutSAF | J9600 | 7.0677 |
|  | OutSAF | J9060 | 6.2445 |
|  | OutSAF | J1450 | 6.0677 |
|  | OutSAF | J2820 | 5.4827 |
|  | OutSAF | J9265 | 5.1932 |
|  | OutSAF | J1110 | 5.0677 |
|  | OutSAF | J9045 | 5.0453 |
|  | OutSAF | J9170 | 4.8197 |
|  | OutSAF | J9250 | 4.7457 |
|  | OutSAF | J9040 | 4.4827 |
|  | OutSAF | J9190 | 4.1932 |
| Level II HCPCS codes\P codes\ | OutSAF | P9038 | 5.4827 |
| Level II HCPCS codes\Q codes\ | OutSAF | Q0180 | 5.7457 |
|  | OutSAF | Q0084 | 4.6210 |
| Medicine\ \Allergy and Clinical Immunology | NCH | 95933 | 4.2601 |
| Medicine\ \Central Nervous System Assessments/Tests (eg, Neuro-Cognitive, Mental Satus, Speech Testing | NCH | 96414 | 5.5584 |
|  | OutSAF | 96530 | 4.7457 |
|  | OutSAF | 96520 | 4.4827 |
|  | NCH | 96412 | 4.4470 |
|  | NCH | 96410 | 4.4019 |
| Medicine\ \General Ophthalmological Services | NCH | 92502 | 7.8044 |
|  | OutSAF | 92605 | 7.0677 |
|  | OutSAF | 92609 | 7.0677 |
|  | OutSAF | 92511 | 6.5441 |
|  | OutSAF | 92612 | 4.0677 |
| Nonoperative intubation and irrigation | OutSAF | 96.08 | 5.0677 |
|  | OutSAF | 96.05 | 5.0677 |
| Nuclear Medicine | OutSAF | 92.12 | 7.0677 |
|  | OutSAF | 92.23 | 7.0677 |
|  | OutSAF | 92.25 | 7.0677 |
|  | MedPAR | 92.29 | 7.0412 |
|  | MedPAR | 92.24 | 6.9302 |
|  | OutSAF | 92.24 | 6.4827 |
|  | OutSAF | 92.29 | 6.4156 |
|  | MedPAR | 92.22 | 6.0677 |
|  | MedPAR | 92.23 | 6.0677 |
|  | OutSAF | 92.19 | 4.4827 |
|  | MedPAR | 92.27 | 4.1488 |
| Operation on nose | MedPAR | 21.22 | 6.0677 |
|  | MedPAR | 21.69 | 5.0677 |
|  | MedPAR | 21.09 | 4.7457 |
| Operation on pharynx | MedPAR | 29.33 | 10.7681 |
|  | MedPAR | 29.12 | 10.1033 |
|  | MedPAR | 29.39 | 9.3896 |
|  | OutSAF | 29.12 | 8.2900 |
|  | MedPAR | 29.53 | 8.0677 |
|  | MedPAR | 29.59 | 7.6526 |
|  | MedPAR | 29.2 | 7.0677 |
|  | OutSAF | 29.11 | 6.7197 |
|  | OutSAF | 29.39 | 6.2603 |
|  | MedPAR | 29.4 | 5.7457 |
|  | MedPAR | 29.11 | 5.4302 |
| Operations on chest wall, pleura, mediastinum, and diaphragm | MedPAR | 34.3 | 5.4827 |
| Operations on cranial and peripheral nerves | MedPAR | 04.5 | 9.6526 |
|  | MedPAR | 04.07 | 6.7457 |
| Operations on esophagus | MedPAR | 42.11 | 7.0677 |
|  | MedPAR | 42.22 | 7.0677 |
|  | MedPAR | 42.25 | 7.0677 |
|  | MedPAR | 42.81 | 6.0677 |
|  | MedPAR | 42.24 | 5.9522 |
|  | OutSAF | 42.23 | 5.5309 |
|  | MedPAR | 42.41 | 5.0677 |
| Operations on external ear | MedPAR | 18.09 | 7.0677 |
|  | MedPAR | 18.6 | 5.0677 |
|  | MedPAR | 18.79 | 4.2603 |
| Operations on eyelids | MedPAR | 08.52 | 9.6526 |
|  | MedPAR | 08.36 | 7.0677 |
|  | MedPAR | 08.70 | 7.0677 |
|  | MedPAR | 08.99 | 7.0677 |
|  | MedPAR | 08.59 | 6.8046 |
|  | MedPAR | 08.89 | 6.0677 |
| Operations on facial bones and joints | MedPAR | 76.31 | 10.5271 |
|  | MedPAR | 76.39 | 10.3896 |
|  | MedPAR | 76.43 | 9.2900 |
|  | MedPAR | 76.91 | 8.6526 |
|  | MedPAR | 76.2 | 8.5271 |
|  | MedPAR | 76.41 | 8.0677 |
|  | MedPAR | 76.92 | 7.3896 |
|  | MedPAR | 76.11 | 7.0677 |
|  | MedPAR | 76.45 | 7.0677 |
|  | MedPAR | 76.46 | 7.0677 |
|  | MedPAR | 76.64 | 7.0677 |
|  | MedPAR | 76.76 | 6.3307 |
|  | MedPAR | 76.93 | 5.4827 |
|  | OutSAF | 76.2 | 5.4827 |
| Operations on lymphatic systems | MedPAR | 40.42 | 10.6526 |
|  | MedPAR | 40.41 | 10.1294 |
|  | MedPAR | 40.40 | 9.8750 |
|  | MedPAR | 40.21 | 7.3896 |
|  | MedPAR | 40.59 | 5.4827 |
|  | MedPAR | 40.3 | 4.3163 |
|  | MedPAR | 40.19 | 4.0677 |
| Operations on muscle, tendon, fascia, and bursa, except hand | MedPAR | 83.32 | 5.7457 |
|  | MedPAR | 83.82 | 5.6691 |
|  | MedPAR | 83.21 | 5.3021 |
|  | MedPAR | 83.43 | 5.0677 |
|  | MedPAR | 83.87 | 5.0677 |
| Operations on nasal sinus | MedPAR | 22.11 | 8.0677 |
|  | MedPAR | 22.02 | 7.0677 |
|  | OutSAF | 22.11 | 5.0677 |
|  | MedPAR | 22.62 | 4.7457 |
|  | MedPAR | 22.2 | 4.0677 |
| Operations on other endocrine glands | MedPAR | 07.62 | 7.3572 |
| Operations on pancreas | MedPAR | 52.14 | 5.0677 |
| Operations on salivary glands and ducts | MedPAR | 26.12 | 9.0677 |
|  | MedPAR | 26.30 | 9.0677 |
|  | OutSAF | 26.12 | 9.0677 |
|  | MedPAR | 26.49 | 8.6526 |
|  | MedPAR | 26.32 | 8.3156 |
|  | MedPAR | 26.29 | 7.8046 |
|  | MedPAR | 26.31 | 7.5333 |
|  | MedPAR | 26.91 | 7.0677 |
|  | OutSAF | 26.49 | 7.0677 |
|  | OutSAF | 26.99 | 7.0677 |
|  | OutSAF | 26.32 | 6.8266 |
|  | OutSAF | 26.91 | 6.0677 |
|  | OutSAF | 26.31 | 5.9522 |
|  | MedPAR | 26.0 | 4.2603 |
|  | OutSAF | 26.0 | 4.2603 |
| Operations on skin and subcutaneous tissue | MedPAR | 86.81 | 7.0677 |
|  | MedPAR | 86.71 | 6.4827 |
|  | MedPAR | 86.70 | 5.9302 |
|  | MedPAR | 86.74 | 5.7930 |
|  | MedPAR | 86.72 | 5.0677 |
|  | MedPAR | 86.69 | 4.9133 |
|  | MedPAR | 86.67 | 4.8453 |
|  | OutSAF | 86.07 | 4.4569 |
| Operations on tongue | MedPAR | 25.2 | 11.3751 |
|  | OutSAF | 25.2 | 11.2376 |
|  | MedPAR | 25.01 | 10.1831 |
|  | MedPAR | 25.4 | 9.6526 |
|  | MedPAR | 25.1 | 9.5912 |
|  | OutSAF | 25.02 | 9.4600 |
|  | MedPAR | 25.59 | 9.3896 |
|  | OutSAF | 25.01 | 9.2197 |
|  | MedPAR | 25.02 | 8.9421 |
|  | MedPAR | 25.3 | 8.6526 |
|  | MedPAR | 25.51 | 7.0677 |
|  | MedPAR | 25.94 | 7.0677 |
|  | OutSAF | 25.09 | 7.0677 |
|  | OutSAF | 25.1 | 6.7628 |
|  | OutSAF | 25.59 | 6.4827 |
| Operations on tonsils and adenoids | OutSAF | 28.11 | 10.2376 |
|  | MedPAR | 28.2 | 9.2376 |
|  | MedPAR | 28.11 | 8.6526 |
|  | MedPAR | 28.92 | 8.6526 |
|  | OutSAF | 28.92 | 8.6526 |
|  | MedPAR | 28.5 | 7.0677 |
|  | MedPAR | 28.99 | 7.0677 |
|  | OutSAF | 28.0 | 7.0677 |
|  | OutSAF | 28.2 | 5.8453 |
| Operations on urethra | MedPAR | 58.22 | 4.4827 |
| Operations onthyroid and parathyroid glands | MedPAR | 06.91 | 7.0677 |
|  | MedPAR | 06.31 | 6.4827 |
|  | MedPAR | 06.09 | 6.0677 |
|  | MedPAR | 06.12 | 6.0677 |
|  | MedPAR | 06.02 | 5.0677 |
| Ophthalmologic and otologic diagnosis and treatment | OutSAF | 95.43 | 7.0677 |
| Other nonoperative procedures | MedPAR | 99.25 | 6.4011 |
|  | MedPAR | 99.74 | 4.6753 |
|  | MedPAR | 99.08 | 4.4827 |
|  | OutSAF | 99.25 | 4.4282 |
|  | MedPAR | 99.28 | 4.1608 |
| Other operations on intestine | OutSAF | 46.32 | 4.3672 |
|  | MedPAR | 46.39 | 4.3373 |
| Other operations on larynx and trachea | MedPAR | 31.75 | 9.0677 |
|  | MedPAR | 31.95 | 8.0677 |
|  | MedPAR | 31.43 | 7.6843 |
|  | OutSAF | 31.43 | 7.2376 |
|  | MedPAR | 31.42 | 6.5882 |
|  | OutSAF | 31.42 | 6.5089 |
|  | MedPAR | 31.41 | 6.0677 |
|  | MedPAR | 31.45 | 6.0677 |
|  | MedPAR | 31.29 | 5.9828 |
|  | MedPAR | 31.5 | 5.4827 |
|  | MedPAR | 31.1 | 5.3683 |
|  | MedPAR | 31.69 | 5.0677 |
|  | MedPAR | 31.44 | 4.4827 |
|  | MedPAR | 31.74 | 4.3672 |
| Other operations on lung and bronchus | OutSAF | 33.23 | 6.2197 |
|  | MedPAR | 33.21 | 4.8977 |
|  | OutSAF | 33.22 | 4.3869 |
|  | MedPAR | 33.23 | 4.0318 |
| Other operations on middle and inner ear | MedPAR | 20.49 | 6.3307 |
|  | MedPAR | 20.01 | 5.6526 |
|  | OutSAF | 20.09 | 5.4827 |
| Other operations on mouth and face | MedPAR | 27.59 | 10.5271 |
|  | MedPAR | 27.49 | 10.1762 |
|  | MedPAR | 27.57 | 9.7681 |
|  | MedPAR | 27.24 | 9.6526 |
|  | MedPAR | 27.55 | 9.3896 |
|  | MedPAR | 27.31 | 9.0677 |
|  | MedPAR | 27.42 | 9.0677 |
|  | OutSAF | 27.22 | 9.0677 |
|  | MedPAR | 27.32 | 8.8750 |
|  | MedPAR | 27.56 | 8.8750 |
|  | MedPAR | 27.22 | 8.6526 |
|  | OutSAF | 27.32 | 8.6526 |
|  | OutSAF | 27.24 | 8.5531 |
|  | OutSAF | 27.56 | 8.2900 |
|  | OutSAF | 27.49 | 8.2900 |
|  | MedPAR | 27.72 | 8.0677 |
|  | OutSAF | 27.59 | 7.4827 |
|  | MedPAR | 27.23 | 7.0677 |
|  | MedPAR | 27.29 | 7.0677 |
|  | MedPAR | 27.99 | 7.0677 |
|  | OutSAF | 27.21 | 7.0677 |
|  | OutSAF | 27.31 | 7.0677 |
|  | OutSAF | 27.42 | 7.0677 |
|  | OutSAF | 27.43 | 6.7390 |
|  | OutSAF | 27.57 | 6.5202 |
|  | MedPAR | 27.0 | 5.8977 |
|  | OutSAF | 27.72 | 5.4827 |
|  | MedPAR | 27.69 | 4.7457 |
|  | OutSAF | 27.23 | 4.0675 |
| Other operations on stomach | OutSAF | 44.39 | 7.0677 |
|  | MedPAR | 44.63 | 5.4827 |
| Other operations on teeth, gums, and alveoli | MedPAR | 24.31 | 9.0677 |
|  | MedPAR | 24.7 | 8.6526 |
|  | OutSAF | 24.11 | 8.6526 |
|  | MedPAR | 24.11 | 7.4827 |
|  | MedPAR | 24.5 | 7.3896 |
|  | MedPAR | 24.12 | 7.0677 |
|  | MedPAR | 24.4 | 7.0677 |
|  | MedPAR | 24.91 | 7.0677 |
|  | OutSAF | 24.12 | 7.0677 |
|  | OutSAF | 24.4 | 6.3307 |
|  | OutSAF | 24.31 | 4.7457 |
|  | OutSAF | 24.5 | 4.3672 |
| Other operations on vessels | MedPAR | 39.91 | 5.4827 |
| Other procedures on musculoskeletal system | MedPAR | 84.05 | 6.0677 |
| Pathology and Laboratory\ \Anatomic Pathology | NCH | 88239 | 7.0675 |
|  | OutSAF | 88321 | 4.7610 |
|  | OutSAF | 88331 | 4.1654 |
| Pathology and Laboratory\ \Immunology | OutSAF | 86337 | 7.0677 |
| Pathology and Laboratory\ \Microbiology | OutSAF | 87420 | 4.4827 |
| Radiology\ \Abdomen | OutSAF | 74350 | 6.1348 |
|  | OutSAF | 74355 | 4.0677 |
| Radiology\ \Chest | OutSAF | 71551 | 7.0677 |
| Radiology\ \Clinical Treatment Planning | NCH | 77399 | 9.0675 |
|  | OutSAF | 77427 | 7.6526 |
|  | OutSAF | 77412 | 7.4213 |
|  | OutSAF | 77408 | 7.4156 |
|  | OutSAF | 77263 | 7.0677 |
|  | NCH | 77777 | 7.0675 |
|  | OutSAF | 77305 | 7.0219 |
|  | OutSAF | 77418 | 6.9828 |
|  | NCH | 77321 | 6.7435 |
|  | NCH | 77305 | 6.7128 |
|  | OutSAF | 77301 | 6.7051 |
|  | NCH | 77310 | 6.6018 |
|  | NCH | 77333 | 6.5736 |
|  | OutSAF | 77321 | 6.5468 |
|  | NCH | 77301 | 6.5346 |
|  | OutSAF | 77310 | 6.5271 |
|  | OutSAF | 77327 | 6.4827 |
|  | OutSAF | 77413 | 6.4289 |
|  | OutSAF | 77333 | 6.3963 |
|  | NCH | 77418 | 6.2279 |
|  | OutSAF | 77315 | 6.1532 |
|  | NCH | 77412 | 6.1499 |
|  | NCH | 77315 | 6.1275 |
|  | NCH | 77413 | 6.0824 |
|  | OutSAF | 77407 | 6.0677 |
|  | OutSAF | 77404 | 6.0677 |
|  | OutSAF | 77334 | 6.0157 |
|  | OutSAF | 77290 | 6.0012 |
|  | OutSAF | 77280 | 5.9866 |
|  | OutSAF | 77417 | 5.9818 |
|  | OutSAF | 77285 | 5.9522 |
|  | NCH | 77334 | 5.8771 |
|  | NCH | 77417 | 5.8768 |
|  | OutSAF | 77336 | 5.8683 |
|  | OutSAF | 77416 | 5.8660 |
|  | OutSAF | 77300 | 5.8020 |
|  | NCH | 77427 | 5.7690 |
|  | OutSAF | 77331 | 5.7655 |
|  | OutSAF | 77402 | 5.7457 |
|  | NCH | 77290 | 5.7277 |
|  | NCH | 77280 | 5.7247 |
|  | NCH | 77263 | 5.6411 |
|  | NCH | 77285 | 5.6129 |
|  | OutSAF | 77295 | 5.6057 |
|  | NCH | 77331 | 5.5913 |
|  | NCH | 77300 | 5.5405 |
|  | NCH | 77416 | 5.5231 |
|  | NCH | 77336 | 5.5183 |
|  | OutSAF | 77409 | 5.4827 |
|  | OutSAF | 77784 | 5.4827 |
|  | NCH | 77470 | 5.4825 |
|  | NCH | 77295 | 5.4437 |
|  | OutSAF | 77403 | 5.4396 |
|  | NCH | 77370 | 5.3736 |
|  | OutSAF | 77470 | 5.3603 |
|  | OutSAF | 77370 | 5.3383 |
|  | OutSAF | 77332 | 5.1857 |
|  | NCH | 77332 | 5.1772 |
|  | NCH | 77761 | 5.0675 |
|  | NCH | 77414 | 5.0675 |
|  | OutSAF | 77414 | 4.9659 |
|  | NCH | 77408 | 4.8975 |
|  | OutSAF | 77783 | 4.2603 |
|  | NCH | 77776 | 4.2601 |
|  | NCH | 77782 | 4.2601 |
|  | NCH | 77262 | 4.0675 |
| Radiology\ \Head and Neck | NCH | 70320 | 6.2279 |
|  | OutSAF | 70355 | 5.3598 |
|  | OutSAF | 70488 | 5.0677 |
|  | NCH | 70355 | 4.6812 |
| Radiology\ \Nuclear Medicine | NCH | 78810 | 5.0675 |
|  | NCH | 78201 | 4.2601 |
| Radiology\ \Other Procedure | OutSAF | 76370 | 5.8385 |
|  | NCH | 76370 | 5.5838 |
|  | NCH | 76400 | 4.4825 |
| Removal and restoration of teeth | MedPAR | 23.6 | 7.0677 |
|  | MedPAR | 23.19 | 6.8612 |
|  | MedPAR | 23.09 | 6.6082 |
|  | OutSAF | 23.09 | 4.4827 |
|  | OutSAF | 23.19 | 4.0677 |
| Repair of cerebral meninges | MedPAR | 02.94 | 4.3128 |
| Replacement and removal of therapeutic appliances | MedPAR | 97.01 | 7.0677 |
|  | MedPAR | 97.23 | 5.7651 |
|  | MedPAR | 97.32 | 5.0677 |
|  | OutSAF | 97.39 | 5.0677 |
|  | MedPAR | 97.39 | 4.4827 |
|  | MedPAR | 97.51 | 4.4432 |
|  | MedPAR | 97.41 | 4.2603 |
| Surgery\ \Lung and Pleura | NCH | 32525 | 5.0675 |
| Surgery\ \Posterior Segment | NCH | 67902 | 7.0675 |
| Surgery\ \Vagina | OutSAF | 57155 | 7.0677 |
| Surgery\Cardiovascular System\Direct Repair of Aneurysm or Excision (Partial or Total) and graft Insertion for Aneurysm, Pseudoaneurysm, Reptured Aneurysm, and Associated Occusive Disease | NCH | 35201 | 5.8451 |
|  | NCH | 35701 | 4.8975 |
|  | NCH | 35261 | 4.7455 |
| Surgery\Cardiovascular System\Lymph Nodes and Lymphatic Channels | NCH | 38724 | 9.1455 |
|  | NCH | 38720 | 8.3894 |
|  | NCH | 38700 | 7.5149 |
|  | NCH | 38542 | 7.4825 |
|  | NCH | 38305 | 5.0675 |
|  | OutSAF | 38999 | 4.4827 |
| Surgery\Cardiovascular System\Portal Decompression Procedures | NCH | 37565 | 7.0675 |
|  | NCH | 37600 | 6.4825 |
|  | NCH | 37605 | 6.0675 |
| Surgery\Digestive system\Anus | OutSAF | 46750 | 5.4827 |
| Surgery\Digestive system\Esophagus | NCH | 43300 | 8.0675 |
|  | NCH | 43496 | 8.0675 |
|  | OutSAF | 43200 | 7.4951 |
|  | OutSAF | 43653 | 7.0677 |
|  | NCH | 43200 | 6.9807 |
|  | OutSAF | 43219 | 5.4827 |
|  | NCH | 43653 | 5.2601 |
|  | OutSAF | 43830 | 5.0677 |
|  | NCH | 43832 | 5.0086 |
|  | OutSAF | 43246 | 4.1651 |
|  | NCH | 43219 | 4.0675 |
| Surgery\Digestive system\Intestine (Except Rectum) | NCH | 44201 | 5.0675 |
|  | NCH | 44130 | 4.6080 |
|  | OutSAF | 44500 | 4.4827 |
|  | OutSAF | 44372 | 4.1608 |
| Surgery\Digestive system\Lips | OutSAF | 40808 | 10.2376 |
|  | OutSAF | 40810 | 9.3896 |
|  | OutSAF | 40510 | 8.9421 |
|  | NCH | 40527 | 8.6524 |
|  | NCH | 40845 | 8.6524 |
|  | OutSAF | 40525 | 8.4827 |
|  | OutSAF | 40816 | 8.0677 |
|  | OutSAF | 40820 | 8.0677 |
|  | OutSAF | 40500 | 8.0677 |
|  | OutSAF | 40520 | 7.6526 |
|  | OutSAF | 40530 | 7.5982 |
|  | OutSAF | 40650 | 7.0677 |
|  | OutSAF | 40652 | 7.0677 |
|  | OutSAF | 40654 | 7.0677 |
|  | OutSAF | 40799 | 7.0677 |
|  | OutSAF | 40812 | 7.0677 |
|  | NCH | 40842 | 7.0675 |
|  | NCH | 40899 | 7.0675 |
|  | NCH | 40814 | 6.8044 |
|  | NCH | 40500 | 6.5529 |
|  | NCH | 40816 | 6.4394 |
|  | NCH | 40525 | 6.3894 |
|  | NCH | 40520 | 6.1240 |
|  | NCH | 40808 | 5.5917 |
|  | NCH | 40812 | 4.7007 |
| Surgery\Digestive system\Salivary Gland and Ducts | OutSAF | 42800 | 10.3896 |
|  | NCH | 42842 | 10.0675 |
|  | NCH | 42890 | 10.0675 |
|  | OutSAF | 42100 | 9.8750 |
|  | NCH | 42844 | 9.3894 |
|  | OutSAF | 42120 | 9.0677 |
|  | NCH | 42870 | 9.0675 |
|  | NCH | 42894 | 9.0675 |
|  | NCH | 42950 | 9.0675 |
|  | NCH | 42802 | 8.8748 |
|  | NCH | 42120 | 8.7304 |
|  | OutSAF | 42802 | 8.6526 |
|  | NCH | 42699 | 8.6524 |
|  | NCH | 42425 | 8.6524 |
|  | OutSAF | 42804 | 8.3896 |
|  | NCH | 42280 | 8.3894 |
|  | NCH | 42426 | 8.2899 |
|  | OutSAF | 42107 | 8.0677 |
|  | OutSAF | 42420 | 8.0677 |
|  | NCH | 42107 | 8.0675 |
|  | NCH | 42200 | 8.0675 |
|  | NCH | 42845 | 8.0675 |
|  | NCH | 42999 | 8.0675 |
|  | NCH | 42892 | 8.0675 |
|  | OutSAF | 42999 | 7.9157 |
|  | NCH | 42800 | 7.8975 |
|  | NCH | 42420 | 7.5080 |
|  | OutSAF | 42500 | 7.0677 |
|  | OutSAF | 42806 | 7.0677 |
|  | OutSAF | 42870 | 7.0677 |
|  | OutSAF | 42106 | 7.0677 |
|  | NCH | 07740 | 7.0675 |
|  | NCH | 42182 | 7.0675 |
|  | NCH | 42325 | 7.0675 |
|  | NCH | 42450 | 7.0675 |
|  | NCH | 42505 | 7.0675 |
|  | OutSAF | 42104 | 6.6526 |
|  | OutSAF | 42808 | 6.4827 |
|  | NCH | 42299 | 6.4825 |
|  | NCH | 42500 | 6.4825 |
|  | NCH | 42808 | 6.4825 |
|  | OutSAF | 42400 | 6.3307 |
|  | NCH | 42100 | 6.3019 |
|  | OutSAF | 42826 | 6.1932 |
|  | NCH | 42660 | 6.0675 |
|  | NCH | 42953 | 6.0675 |
|  | NCH | 42440 | 6.0675 |
|  | OutSAF | 42410 | 5.8453 |
|  | OutSAF | 42415 | 5.6526 |
|  | NCH | 42160 | 5.4825 |
|  | NCH | 42106 | 4.9800 |
|  | OutSAF | 42440 | 4.8977 |
| Surgery\Digestive system\Tongue and Floor of Mouth | OutSAF | 41120 | 10.9745 |
|  | NCH | 41150 | 10.3894 |
|  | NCH | 41135 | 10.3405 |
|  | NCH | 41155 | 10.3154 |
|  | NCH | 41130 | 9.8748 |
|  | OutSAF | 41116 | 9.2376 |
|  | OutSAF | 41105 | 9.1831 |
|  | NCH | 41120 | 9.1484 |
|  | OutSAF | 41100 | 9.0677 |
|  | NCH | 41145 | 9.0675 |
|  | NCH | 41599 | 9.0675 |
|  | OutSAF | 41108 | 8.7681 |
|  | OutSAF | 41130 | 8.6526 |
|  | NCH | 41153 | 8.6524 |
|  | OutSAF | 41114 | 8.0677 |
|  | OutSAF | 41110 | 7.8046 |
|  | NCH | 41116 | 7.4394 |
|  | NCH | 41105 | 7.3570 |
|  | OutSAF | 41827 | 7.0677 |
|  | OutSAF | 41113 | 7.0677 |
|  | NCH | 41140 | 7.0675 |
|  | NCH | 41252 | 7.0675 |
|  | NCH | 41827 | 7.0675 |
|  | NCH | 41108 | 7.0675 |
|  | NCH | 41113 | 6.9419 |
|  | OutSAF | 41112 | 6.6526 |
|  | OutSAF | 41826 | 6.4827 |
|  | NCH | 41100 | 6.4708 |
|  | OutSAF | 41830 | 6.0677 |
|  | NCH | 41000 | 6.0675 |
|  | NCH | 41874 | 5.7873 |
|  | NCH | 41826 | 5.5820 |
|  | OutSAF | 41825 | 5.4827 |
|  | NCH | 41112 | 5.3596 |
|  | NCH | 41825 | 5.3305 |
|  | NCH | 41250 | 5.0675 |
|  | NCH | 41899 | 5.0350 |
| Surgery\Intergumentary system\Repair (Closure) | NCH | 12057 | 7.0675 |
| Surgery\Intergumentary system\Skin Replacement Surgery and Skin Substitues | NCH | 15758 | 9.8748 |
|  | NCH | 15757 | 9.7679 |
|  | OutSAF | 15770 | 7.0677 |
|  | OutSAF | 15620 | 7.0677 |
|  | NCH | 15840 | 6.4825 |
|  | NCH | 15756 | 5.7455 |
|  | NCH | 15732 | 5.3937 |
|  | NCH | 15120 | 5.0935 |
|  | NCH | 15620 | 4.8975 |
|  | NCH | 15350 | 4.8975 |
|  | OutSAF | 15630 | 4.7457 |
|  | NCH | 15734 | 4.2790 |
|  | OutSAF | 15851 | 4.2603 |
|  | NCH | 15200 | 4.2601 |
| Surgery\Musculoskeletal System\General | NCH | 20969 | 9.2374 |
|  | NCH | 20955 | 8.0675 |
|  | NCH | 20970 | 7.0675 |
| Surgery\Musculoskeletal System\Head | NCH | 21080 | 9.8748 |
|  | NCH | 21244 | 9.4825 |
|  | NCH | 21044 | 9.3894 |
|  | NCH | 21079 | 9.1829 |
|  | NCH | 21082 | 8.6524 |
|  | NCH | 21198 | 8.3894 |
|  | NCH | 21076 | 8.2899 |
|  | OutSAF | 21044 | 8.0677 |
|  | NCH | 21081 | 8.0675 |
|  | NCH | 21070 | 7.0675 |
|  | NCH | 21193 | 7.0675 |
|  | NCH | 21196 | 7.0675 |
|  | NCH | 21089 | 6.8264 |
|  | NCH | 21215 | 6.6524 |
|  | NCH | 21299 | 6.2601 |
|  | NCH | 21249 | 6.0675 |
|  | NCH | 21032 | 5.4825 |
|  | NCH | 21085 | 5.0675 |
|  | NCH | 21110 | 4.2601 |
| Surgery\Nervous System\Endocrine System | NCH | 60200 | 4.8451 |
| Surgery\Nervous System\Extracranial Nerves, Peripheral Nerves, and Autonomic Nervous System | NCH | 64886 | 8.6524 |
|  | NCH | 64716 | 8.0675 |
|  | NCH | 64885 | 8.0675 |
|  | NCH | 64740 | 7.0675 |
|  | NCH | 64742 | 7.0675 |
|  | NCH | 64902 | 7.0675 |
| Surgery\Nervous System\Skull, Meninges, and Brain | NCH | 61576 | 7.0675 |
|  | NCH | 61590 | 7.0675 |
|  | NCH | 61580 | 5.4825 |
|  | NCH | 61626 | 4.6080 |
| Surgery\Nervous System\Spine and SpiralCord | OutSAF | 62365 | 6.0677 |
| Surgery\Ocular/Auditory System\Auditory System | NCH | 69720 | 7.0675 |
|  | NCH | 69502 | 7.0675 |
|  | NCH | 69535 | 5.4825 |
|  | OutSAF | 69220 | 4.0677 |
| Surgery\Respiratory System\Accessory Sinuese | NCH | 31365 | 9.3894 |
|  | OutSAF | 31535 | 8.7636 |
|  | OutSAF | 31510 | 8.6526 |
|  | NCH | 31395 | 8.6524 |
|  | NCH | 31390 | 8.0675 |
|  | NCH | 31360 | 8.0675 |
|  | NCH | 31526 | 7.8748 |
|  | NCH | 31535 | 7.8107 |
|  | OutSAF | 31525 | 7.5113 |
|  | OutSAF | 31515 | 7.0677 |
|  | NCH | 31367 | 7.0675 |
|  | NCH | 31368 | 7.0675 |
|  | NCH | 31370 | 7.0675 |
|  | NCH | 31560 | 7.0675 |
|  | OutSAF | 31536 | 6.8225 |
|  | OutSAF | 31576 | 6.3896 |
|  | NCH | 31576 | 6.2601 |
|  | OutSAF | 31830 | 6.0677 |
|  | OutSAF | 31575 | 5.9448 |
|  | NCH | 31515 | 5.6889 |
|  | NCH | 31525 | 5.5139 |
|  | OutSAF | 31899 | 5.4827 |
|  | NCH | 31599 | 5.4825 |
|  | NCH | 31530 | 5.0675 |
|  | NCH | 31540 | 4.9520 |
|  | OutSAF | 31622 | 4.5691 |
|  | NCH | 31613 | 4.4825 |
|  | NCH | 31588 | 4.2601 |
|  | NCH | 31600 | 4.2443 |
|  | OutSAF | 31599 | 4.0677 |

*ICD-9-CM procedure codes of format XX.XX; CPT procedure codes of format XXXXX; clinical categories based on ICD-9-CM and CPT coding manuals and from http://www.cms.gov/Medicare/Coding/HCPCSReleaseCodeSets/Alpha-Numeric-HCPCS-Items/CMS1253559.html
